# News and Coming Events

**Published:** 1908-12-26

**Authors:** 


### NEWS AND COMING EVENTS.

A VERY successful inaugural meeting of the York branch of the Research Defence Society was held on December 11 under the chairmanship of Mr. W. F. H. Thompson, J.P., when a lecture was given by Mr. Stephen Paget, F.R.C.S., on the work and objects of the Society.

THE Home Secretary has appointed a departmental committee to inquire into the law relating to coroners and coroners' inquests, and into the practice in coroners' courts. The Chairman of the committee is Sir Mackenzie Chalmers, K.C.B., C.S.I., and the other members are: Sir Malcolm Morris, K.C.V.O., F.R.C.S., Sir Horatio Shephard, T. A. Bramsdon, Esq., M.P., William H. Willcox, Esq., M.D., etc. The Secretary to the committee is J. F. Moylan, Esq., of the Home Office.

THE Poisons and Pharmacy Bill, as amended in the Standing Committee, was considered in the House of Commons on December 17, when Mr. Herbert Samuel moved an amendment postponing the date at which the Bill will come into operation from January 1 to April 1, 1909. The amendment was agreed to, and the Bill was then read the third time.

IN the House of Commons on December 15, in answer to a question by Mr. Kettle, the Under Secretary for India stated that the professors of the Medical College and the physicians and surgeons of the General Hospital, Madras, are at present officers of the Indian Medical Service, which is recruited for both military and civil duty. Medical officers outside that service are not shut off from higher appointments, although in practice they seldom attain the highest posts. The Secretary of State does not consider the present condition of the matter to be satisfactory, and is in communication with the Government of India as to the desirability of promoting the growth of an independent medical profession in India and of extending the employment of civil medical practitioners recruited in India.

THE Paddington Green Children's Hospital Committee earnestly appeal for funds to enable them to provide for the out-patient department increased accommodation, rendered necessary owing to the growth in the number of patients applying for relief. The extension has, in fact, too long been deferred through lack of funds. The present out-patient department was built in 1888, since which time its work has steadily increased. The Medical Committee have for some time urged upon the Committee of Management the need of additional undressing, consulting, anæsthetic, and operating rooms, more waiting-room accommodation, and especially an anæsthetic recovery room. Included in the proposed extension will be a covered way for patients awaiting admission to the waiting hall and for perambulators. The hospital has already acquired the necessary freehold land adjoining the building, and plans are being prepared. The total cost is estimated at £5,000.

THE HOSPITAL DECEMBER 26, 1908.

